# Silver Nanoparticles Synthesized from *Abies alba* and *Pinus sylvestris* Bark Extracts: Characterization, Antioxidant, Cytotoxic, and Antibacterial Effects

**DOI:** 10.3390/antiox12040797

**Published:** 2023-03-24

**Authors:** Irina Macovei, Simon Vlad Luca, Krystyna Skalicka-Woźniak, Cristina Elena Horhogea, Cristina Mihaela Rimbu, Liviu Sacarescu, Gabriela Vochita, Daniela Gherghel, Bianca Laura Ivanescu, Alina Diana Panainte, Constantin Nechita, Andreia Corciova, Anca Miron

**Affiliations:** 1 Faculty of Pharmacy, Grigore T. Popa University of Medicine and Pharmacy, 700115 Iasi, Romania; 2 Biothermodynamics, TUM School of Life Sciences, Technical University of Munich, D-85354 Freising, Germany; 3 Department of Natural Products Chemistry, Medical University of Lublin, 20-093 Lublin, Poland; 4 Department of Public Health, Ion Ionescu de la Brad University of Life Sciences, 700489 Iasi, Romania; 5 Petru Poni Institute of Macromolecular Chemistry, 700487 Iasi, Romania; 6 NIRDBS, Institute of Biological Research Iasi, 700107 Iasi, Romania; 7 Marin Dracea National Institute for Research and Development in Forestry, 725100 Campulung Moldovenesc, Romania

**Keywords:** phytofunctionalized silver nanoparticles, bark extract, *Abies alba* Mill., *Pinus sylvestris* L., A-375 human melanoma cells, DPPH scavenging activity, ABTS scavenging activity, antibacterial activity

## Abstract

In recent years, phytofunctionalized AgNPs have attracted great interest due to their remarkable biological activities. In the present study, AgNPs were synthesized using *Abies alba* and *Pinus sylvestris* bark extracts. The chemical profile of these bark extracts was analyzed by LC-HRMS/MS. As a first step, the synthesis parameters (pH, AgNO_3_ concentration, ratio of bark extract and AgNO_3_, temperature, and reaction time) were optimized. The synthesized AgNPs were characterized by ATR-FTIR spectroscopy, DLS, SEM, EDX, and TEM. Their antioxidant, cytotoxic, and antibacterial properties were evaluated by the DPPH, ABTS, MTT, and broth microdilution assays, respectively. *Abies alba* and *Pinus sylvestris* bark extract-derived AgNPs were well-dispersed, spherical, small (average particle size of 9.92 and 24.49 nm, respectively), stable (zeta potential values of −10.9 and −10.8 mV, respectively), and cytotoxic to A-375 human malignant melanoma cells (IC_50_ = 2.40 ± 0.21 and 6.02 ± 0.61 μg/mL, respectively). The phytosynthesized AgNPs also showed antioxidant and antibacterial effects.

## 1. Introduction

Among nanomaterials, silver nanoparticles (AgNPs) have gained increasing attention over the last years due to their strong antimicrobial activity and unique electrical, optical, and catalytic properties. Nowadays, AgNPs have been imposed as a desirable nanomaterial in different fields such as medicine (impregnation of catheters, meshes for wounds and tissue therapy, bone and cardiovascular implants, bio-imaging, bio-sensing), cosmetics, food preservation, environmental protection (water disinfection), paint coating industry, and catalysis of various processes [1]. Many of the biomedical applications of AgNPs are based on their broad-spectrum antimicrobial activity against bacteria, fungi, and viruses [2]. AgNPs exert higher toxicity against Gram-negative bacteria which is strongly related to the thinner peptidoglycan layer in the bacterial cell wall [3,4]. AgNPs are also active against drug-resistant bacteria, enhance the antibacterial activity of antibiotics [4], have antitumor properties, inhibit angiogenesis, and increase chemosensitivity in multidrug-resistant cancer cells [5,6]. 

The bioactivity of AgNPs can be enhanced and/or enlarged by phytofunctionalization. This can be achieved by using plant extracts to synthesize AgNPs. Phytochemicals present in plant extracts have multiple functions: they reduce Ag^+^ to metallic Ag and stabilize and modulate the activity of AgNPs [7,8]. The use of the aqueous extracts of *Hemidesmus indicus* roots and *Mikania cordata* leaves in the synthesis of AgNPs resulted in strong antibacterial activity against Gram-positive *Staphylococcus aureus* and potent cytotoxicity against PA-1 ovarian cancer cell line [9,10]. AgNPs synthesized using the soap nuts (*Sapindus* sp.) aqueous extract showed higher toxicity against Gram-positive bacteria (*S. aureus*) than Gram-negative bacteria (*Escherichia coli*, *Pseudomonas aeruginosa*) [11]. Phytofunctionalized AgNPs, active against both Gram-positive and Gram-negative bacteria, were obtained using extracts of *Diospyros paniculata* and *Diospyros sylvatica* roots, *Acorus calamus* rhizome, *Cucurbita maxima* petals, *Moringa oleifera*, *Azadirachta indica*, *Sesbania grandiflora*, *Ginkgo biloba*, *Carica papaya*, and *Nigella arvensis* leaves, *Coffea arabica* seeds, and cacao [2,12]. Phytofunctionalized AgNPs have also noticeable antioxidant effects conferred by both elemental Ag and phytochemicals capping AgNPs, the plant extract concentration and antioxidant potential of phytofunctionalized AgNPs being positively correlated [13]. AgNPs synthesized using the aqueous extract of *Allium cepa* bulbs showed promising effects for the treatment of diabetes: inhibition of enzymes involved in carbohydrate metabolism (α-amylase, α-glucosidase) and antioxidant activity but low cytotoxicity against 3T3 pre-adipocytes [14]. AgNPs obtained using the ethanolic extract of *Tabernaemontana divaricata* leaves prevented sodium selenite-induced cataractogenesis in in vitro and in vivo models. In vitro AgNPs attenuated the oxidative stress (decrease in reduced glutathione and activity of antioxidant enzymes, increase in malondialdehyde) in lenses exposed to sodium selenite, thus impeding lens opacification [15]. In selenite-challenged Wistar rat pups, the same AgNPs prevented cataractogenesis by maintaining calcium homeostasis (regulation of lenticular ATPases activity, regulation of genes encoding calcium transporter proteins) which is essential for the integrity of lenticular structural and cytoskeletal proteins. In the same study, chemically synthesized AgNPs showed insignificant anticataractogenic effects [16]. AgNPs synthesized using an aqueous extract of *Turbinaria conoides*, a brown seaweed, showed heart and liver regeneration effects in zebrafish (*Danio rerio*) as revealed by histological studies [17]. Overall, in contrast to “naked” AgNPs, the phytofunctionalized ones have important advantages such as improved bioactivity, higher stability, and reduced toxicity [18].

Coniferous plants, consisting of 8 families, 70 genera, and 630 species distributed worldwide, represent an important source of bioactive phytochemicals (polyphenols, terpenoids, alkaloids) and therefore, they are good candidates for the phytosynthesis of AgNPs [19,20]. Extracts obtained from different parts of coniferous trees (bark, needles, cones, twigs, heart wood, berries) were reported to exhibit various biological effects (antioxidant, anti-inflammatory, anticancer, antimicrobial, analgesic, antidiabetic, anticonvulsant), some extracts being commercially available on the market [20]. 

*Abies alba* Mill. (Pinaceae, silver fir) and *Pinus sylvestris* L. (Pinaceae, Scots pine) are important timber trees in Romania, the bark being the main by-product of the industrial processing of the wood [21,22]. Previous investigations on *A. alba* bark extracts in cell-free and cell-based assays revealed antioxidant (free radical scavenging activity, decrease of the production of reactive oxygen species, reduction of lipoprotein oxidation) [22,23,24], antisteatotic (decrease of lipid and cholesterol accumulation in hepatocytes, slight induction of cholesterol conversion to bile acids), and antihypertensive (inhibition of angiotensin-converting enzyme) effects [24]. *P. sylvestris* bark extracts were reported to display anti-inflammatory activity (reduction of the synthesis of pro-inflammatory prostaglandin E_2_ and nitric oxide) in murine macrophages [25], pro-apoptotic effects in human cervical adenocarcinoma (HeLa) cells [21], antiparasitic activity against *Cryptosporidium parvum*, responsible for severe diarrhoeal disease [26], antibacterial activity against *S. aureus*, including a methicillin-resistant strain, and free radical scavenging activity [27]. To the best of our knowledge, there is no report on the phytosynthesis of AgNPs from *A. alba* or *P. sylvestris* bark extracts. Therefore, this study was designed to synthesize AgNPs by using the aqueous extracts of *A. alba* and *P. sylvestris* bark, to characterize the synthesized AgNPs, and to investigate their antioxidant, cytotoxic, and antibacterial properties. 

## 2. Materials and Methods

### 2.1. Plant Material

*A. alba* and *P. sylvestris* bark fragments were collected from mature trees (35–40 years old, 18–25 cm circumference at breast height) in Suceava county (47°45′13” N, 26°12′19” E), Romania, in March 2018. Species authentication was performed by Dr. Constantin Nechita (Forest Research and Management Institute, Campulung Moldovenesc, Suceava, Romania). Voucher specimens (AA1520/2018, PS1517/2018) are deposited in the Department of Pharmacognosy-Phytotherapy, Faculty of Pharmacy, Grigore T. Popa University of Medicine and Pharmacy, Iasi, Romania. Bark pieces were washed with distilled and then ultra-pure water, chopped, dried in a dark room at 20 ± 2 °C, powdered, and stored at +4 °C until extraction. 

### 2.2. Chemicals and Reagents 

Ciprofloxacin hydrochloride, Folin–Ciocalteu’s phenol reagent, gallic acid, sodium carbonate, formic acid (HPLC), acetonitrile (HPLC), 2,2-diphenyl-1-picrylhydrazyl (DPPH) radical, 2,2′-azinobis(3-ethylbenzothiazoline-6-sulfonic acid) diammonium salt (ABTS), (R)-(+)-6-hydroxy-2,5,7,8-tetramethylchroman-2-carboxylic acid (Trolox), silver nitrate (AgNO_3_), and dimethyl sulfoxide (DMSO) were purchased from Sigma-Aldrich (Steinheim, Germany). 3-(4,5-Dimethyl-2-thiazolyl)-2,5-diphenyl-2H-tetrazolium bromide (MTT) was obtained from Merck (Darmstadt, Germany). Dulbecco’s Modified Eagle’s Medium/Dulbecco’s Minimum Essential Media (DMEM) and penicillin-streptomycin solution were purchased from Biological Industries Israel Beit Haemek Ltd. (Beit Haemek, Israel). Mueller-Hinton broth was supplied by Oxoid (Basingstoke, UK). Fetal bovine serum and trypsin/ethylenediaminetetraacetic acid (EDTA) solution were provided by Biochrom (Berlin, Germany). All other chemicals and reagents were of analytical grade. Ultra-pure water was obtained from SG Water Ultra Clear TWF water purification system (Barsbüttel, Germany). 

### 2.3. Microorganisms

*Staphylococcus aureus* ATCC 25923 and ATCC 43300, *Staphylococcus epidermidis* ATCC 12228, *Escherichia coli* ATCC 25922, and *Pseudomonas aeruginosa* ATCC 9027 were purchased from the American Type Culture Collection (ATCC, Manassas, VA, USA).

### 2.4. Cell Lines

African green monkey kidney (Vero, ATCC^®^ CCL-81™) and human malignant melanoma (A-375, ATCC^®^ CRL-1619™) cell lines were procured from ATCC (Manassas, VA, USA). 

### 2.5. Preparation of Bark Extracts

Extraction was performed with ultra-pure water at a ratio of 1:10 as previously described. Briefly, bark powder (10 g) was mixed with ultra-pure water (100 mL) at 60 °C followed by 3 h stirring at room temperature, filtration, and addition of ultra-pure water to 100 mL final volume [28,29]. 

### 2.6. Chemical Characterization of Bark Extracts

#### 2.6.1. Qualitative Analysis of Bark Extracts

The chemical profile of bark extracts was analyzed by liquid chromatography hyphenated with high-resolution tandem mass spectrometry (LC-HRMS/MS). The analysis was performed on an Agilent 1200 HPLC system (Agilent Technologies, Santa Clara, California, USA) equipped with auto-sampler (G1329B), degasser (G1379B), binary pump (G1312C), thermostat (G1316A), diode array detector (G1315D), Agilent ESI-Q-TOF mass spectrometer (G6530B), nitrogen generator (Parker Hannifin Corp., Cleveland, OH, USA), and compressed air generator (Jun-Air Oxymed, Łódź, Poland). The chromatographic separations were performed as previously described [29,30] with minor changes: column: Phenomenex Gemini C18 (2 × 100 mm, 3 μm), column temperature 20 °C, mobile phase: 0.1% formic acid in water (A) and 0.1% formic acid in acetonitrile (B), elution gradient: 0 min: 5% B, 0–45 min: 5–60% B, 45–55 min: 60–95% B, flow rate 0.2 mL/min, and injection volume: 10 μL. The following MS parameters were used: negative ionization mode, *m/z* range 70–1000, gas (N_2_) temperature 275 °C, N_2_ flow 10 L/min, nebulizer 35 psi, sheath gas temperature 325 °C, sheath gas flow rate 12 L/min, capillary voltage 4000 V, nozzle voltage 1000 V, skimmer 65 V, fragmentor 140 V, fixed collision-induced dissociation energies 10 and 30 V. MassHunter Qualitative Analysis Navigator B.08.00 software (Agilent) was used for data processing. 

#### 2.6.2. Quantitative Analysis of Bark Extracts 

The total phenolic content in bark extracts was quantified using the Folin–Ciocalteu method. In summary, the reaction mixture consisted of a sample (0.04 mL), ultra-pure water (3.16 mL), Folin–Ciocalteu’s phenol reagent (0.2 mL), and 20% sodium carbonate (0.6 mL). After 2 h incubation at room temperature, the absorbance was determined at 765 nm (Specord 210 Plus spectrophotometer, Analytik Jena, Jena, Thuringia, Germany). The phenolic content was expressed as gallic acid equivalents (μg/mL) [31,32]. The assay was carried out in triplicate. 

The total phenolic content was also determined in the reaction mixtures (bark aqueous extract and 2 mM AgNO_3_, 1:9, *v*/*v*) before and after the synthesis and separation of AgNPs.

### 2.7. Synthesis of AgNPs

The synthesis of AgNPs was monitored and optimized by recording the UV-Vis spectra of the reaction mixtures in the wavelength range of 350–800 nm [29,33,34,35,36] on a Specord 210 Plus spectrophotometer (Analytik Jena, Jena, Thuringia, Germany). In order to obtain the maximum yield of AgNPs, the synthesis was conducted at various parameters such as pH value of the reaction mixture (2, 4, 6, 8, and 10), AgNO_3_ concentration (0.5, 1, 2, and 3 mM), ratio of aqueous bark extract and AgNO_3_ (1:9, 2:8, 5:5, 7:3, and 9:1, *v*/*v*), temperature (30, 40, 50, 60, 70, and 80 °C), and reaction time (10, 20, 30, 60, and 120 min). HCl (0.1 N) and NaOH (0.1 N) were used to adjust the pH of the reaction mixture. Once the optimization studies were completed, the synthesis of AgNPs was conducted using the optimal parameters. The dispersed AgNPs were further separated by centrifugation at 9000 rpm for 20 min (Hettich Rotina 380 R centrifuge, Hettich, Tuttlingen, Germany), washed 5 times with ultra-pure water to remove the unreacted material, and finally freeze-dried (Unicryo TFD 5505 freeze dryer, UniEquip GmbH, Munich, Germany). The AgNPs pellets were stored at +4 °C until use. 

### 2.8. Characterization of AgNPs

AgNPs were characterized by attenuated total reflection Fourier-transform infrared (ATR-FTIR) spectroscopy, dynamic light scattering (DLS), scanning electron microscopy (SEM), energy dispersive X-ray analysis (EDX), and transmission electron microscopy (TEM). The procedures were carried out as previously described [29], with some changes. The functional groups on the surface of AgNPs, belonging to the compounds in bark extracts acting as capping/stabilizing agents, were detected by ATR-FTIR spectroscopy (Bruker Alpha-P ATR FTIR spectrometer, Bruker, Ettlingen, Germany). In brief, the aqueous bark extracts (30 mL of each) were concentrated under reduced pressure at 40 °C (Büchi R-210 rotary evaporator system, Büchi Labortechnik AG, Flawil, Switzerland) and freeze-dried (Unicryo TFD 5505 freeze dryer, UniEquip GmbH, Munich, Germany). Freeze-dried extracts and AgNPs pellets were subjected to ATR-FTIR spectroscopy in the spectral region of 4000–400 cm^−1^ with a resolution of 4 cm^−1^. The size and morphology of AgNPs were investigated by SEM coupled with EDX and TEM. For SEM-EDX measurements, a Quanta 200 scanning electron microscope equipped with an energy dispersive spectrometer (FEI Company, Hillsboro, OR, USA) was used. AgNPs pellets were analyzed in low vacuum mode at an operating voltage of 10 kV. TEM analyses, operated at 100 keV, were performed using a Hitachi High-Tech HT7700 transmission electron microscope (Hitachi High-Technologies Corporation, Tokyo, Japan) equipped with a Bruker EDX detector. Prior to analysis, colloidal AgNPs solutions (a drop of each) were placed on carbon-coated copper grids (300-mesh) and subjected to 24 h vacuum-drying (room temperature). DLS measurements were made on a Malvern Zetasizer Nano-ZS (Malvern Instruments, Malvern, UK). The hydrodynamic diameters and polydispersity index (PDI) values of the colloidal AgNPs solutions were determined using He/Ne laser (λ = 633 nm); electrophoretic light scattering was used to measure zeta potential. The colloidal AgNPs solutions refer to the reaction mixtures in which the synthesis of AgNPs was completed (before separation of AgNPs by centrifugation). 

### 2.9. Assessment of Antioxidant Activity

#### 2.9.1. DPPH Radical Scavenging Assay

The antioxidant potential of synthesized AgNPs was initially evaluated by the DPPH radical scavenging assay [37]. A volume of 1800 μL of 0.002% DPPH solution in methanol was mixed with 200 μL of various dilutions of AgNPs in ultra-pure water (50–500 μg/mL). After 30 min incubation at room temperature in dark, the absorbance was determined at 515 nm (Specord 210 Plus spectrophotometer, Analytik Jena, Jena, Thuringia, Germany). Trolox (50–500 μg/mL) was used as positive control. The DPPH radical scavenging activity (%) was calculated as follows: 100 × (A_C_ − A_S_)/A_C_, where A_C_ and A_S_ are the absorbances of the control (DPPH solution) and sample (AgNPs, Trolox), respectively. The experiments were carried out in triplicate.

#### 2.9.2. ABTS Radical Cation Scavenging Assay

The ABTS radical cation was generated by incubating ABTS (7 mM) with potassium persulfate (2.54 mM) for 16 h in dark at room temperature; the solution was further diluted with ethanol to an absorbance of 0.70 ± 0.02 at 734 nm (Specord 210 Plus spectrophotometer, Analytik Jena, Jena, Thuringia, Germany). In brief, the ABTS radical cation solution (1500 μL) was mixed with different concentrations (50–500 μg/mL) of AgNPs/Trolox (positive control). The absorbance was recorded at 734 nm after 6 min incubation at room temperature [31,38]. ABTS radical cation scavenging activity was calculated as described in Section 2.9.1. The assay was performed in triplicate.

### 2.10. Assessment of Cell Toxicity

#### 2.10.1. Cell Cultures

Vero and A-375 cells were cultured in DMEM supplemented with 2% (Vero cells) and 10% (A-375 cells) fetal bovine serum and antibiotics (100 μg/mL penicillin, 100 IU/mL streptomycin) and placed in an incubator at 37 °C in humidified atmosphere (5% CO_2_, 95% air) until an optimal cell confluence (at least 90%) was reached [39]. 

#### 2.10.2. Assessment of Cell Viability

*A. alba* and *P. sylvestris* bark extract-derived AgNPs were applied at different concentrations ranging from 3.125 to 25 µg/mL, for 24 and 48 h. Cell viability was evaluated using the MTT assay as previously reported [40,41], with minor changes. Briefly, the cells were detached with trypsin/EDTA, counted, resuspended in 96-well microplates (7 × 10^3^ cells/well), and maintained in the conditions mentioned above (Section 2.10.1). Once the monolayer formed (24 h), the cells were treated with *A. alba* or *P. sylvestris* bark extract-derived AgNPs. After 24/48 h treatment, the medium was removed, and the cells were washed with phosphate-buffered saline, followed by the addition of fresh growth medium (100 μL/well) and MTT (5 mg/mL, 10 μL/well) and incubation for 3 h at 37 °C (5% CO_2_, 95% air). DMSO (100 μL/well) was further added to solve the insoluble formazan crystals generated after MTT reduction [42,43,44]. The absorbance of the formazan dye was quantified at 570 nm using a microplate reader (Biochrom, Berlin, Germany). The cell viability (%) was calculated as follows: A_S_/A_C_ × 100, where A_S_ and A_C_ are the absorbances of the formazan dye produced in samples (treated cells) and control (untreated cells), respectively. The experiments were performed in triplicate. 

#### 2.10.3. Assessment of Cell Morphology

Morphological changes in Vero and A-375 cells after 24 and 48 h treatment were examined using a Nikon Eclipse TS100 inverted microscope (Nikon, Tokyo, Japan) equipped with a digital camera (MSHOT MS60).

### 2.11. Assessment of Antibacterial Activity

The antibacterial activity of AgNPs was evaluated using the broth microdilution method as previously described [45,46], with slight modifications. Prior to the experiment, stock solutions of AgNPs and ciprofloxacin were prepared (*A. alba* and *P. sylvestris* bark extract-derived AgNPs: 250 μg/mL, ciprofloxacin: 2560 μg/mL) and further subjected to two-fold serial dilutions. AgNPs and ciprofloxacin stock solutions and dilutions were prepared in sterile ultra-pure water. In brief, 30 μL of 0.5 McFarland standard bacterial inoculum was mixed with two-fold serial dilutions of AgNPs (30 μL) or antibiotic (10 μL) and Mueller-Hinton broth up to 200 μL; the mixtures were incubated for 24 h at 37 °C followed by an evaluation of bacterial growth. The minimum inhibitory concentration (MIC) value was the lowest concentration of an antibacterial agent that completely inhibited the growth of bacteria [46,47]. 

### 2.12. Data Analysis

The total phenolic content was expressed as mean ± standard deviation (SD). The antioxidant activity was expressed as EC_50_ values (half maximal effective concentration) calculated by linear interpolation between the values above and below 50% activity; the results were expressed as mean ± SD. The cytotoxic activity was expressed as IC_50_ values (concentration of sample producing 50% inhibition of cell viability) using the polynomial graphic plots by modeling the percentage of cytotoxicity vs. concentration of AgNPs; the results were expressed as mean ± standard error (SE). The paired *t*-test was used to assess the differences between samples and control. 

## 3. Results

### 3.1. Chemical Characterization of Bark Extracts

The results are presented in the Supplementary File (Supplementary Figure S1, Supplementary Table S1). 

### 3.2. Optimization of Synthesis Parameters

**Figure 1:**
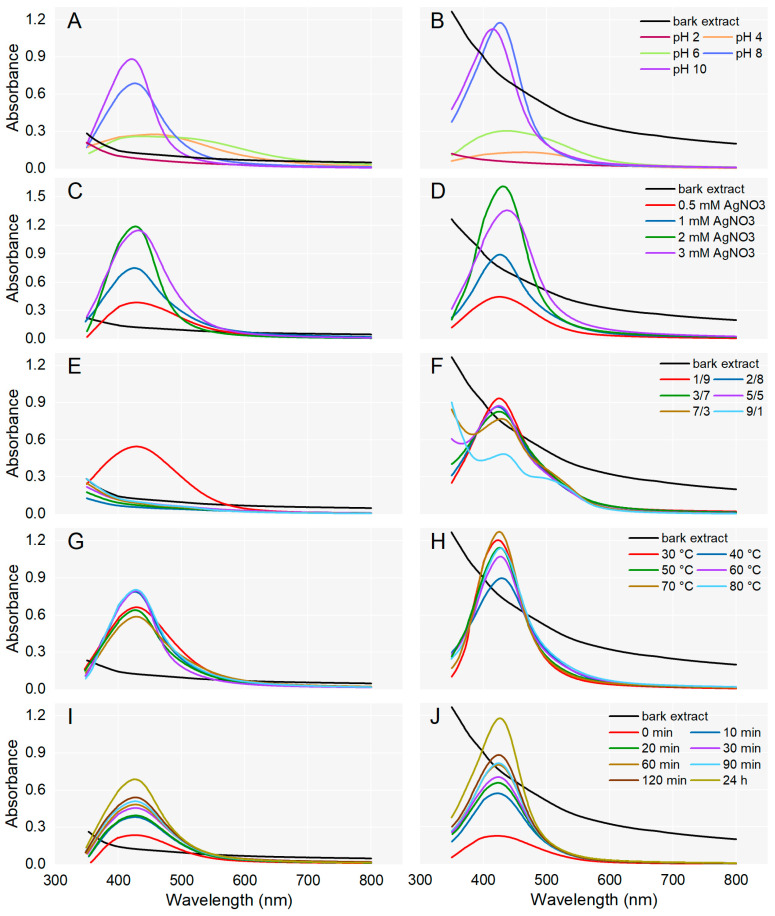
Effect of pH, AgNO_3_ concentration, ratio between the volumes of plant extract and AgNO_3_, temperature, and reaction time on the synthesis of AgNPs from *Abies alba* (**A**,**C**,**E**,**G**,**I**) and *Pinus sylvestris* (**B**,**D**,**F**,**H**,**J**) bark aqueous extracts.

For the synthesis of AgNPs, *A. alba*/*P. sylvestris* bark aqueous extract was mixed with AgNO_3_ under continuous stirring. Different parameters (pH, AgNO_3_ concentration, ratio between the volume of bark extract and AgNO_3_, temperature, and reaction time) were optimized for the synthesis of AgNPs which was monitored by UV-Vis spectroscopy (Figure 1). The generation of AgNPs was indicated by a color change and a characteristic absorption band (Surface Plasmon Resonance (SPR) band) in the visible domain (400–500 nm), caused by the collective oscillations of the free electrons at the surface of metallic Ag when excited by light [48]. 

To evaluate the effect of pH on AgNPs synthesis, the process was conducted at various pH values (2, 4, 6, 8, and 10). For both bark extracts, the synthesis of AgNPs could not be achieved at acidic pH (pH 2). At pH 4 and 6, slightly prominent bands between 400 and 500 nm indicated that the synthesis of AgNPs was initiated. At basic pH (8 and 10), characteristic SPR peaks were observed in the region of 410–430 nm, supporting the formation of AgNPs. In the case of both bark extracts, at pH 10, SPR peaks were sharper and their absorption maxima slightly shifted to shorter wavelengths (Figure 1A,B) suggesting the formation of smaller-sized AgNPs [49]. A single sharp SPR band at short wavelengths indicates the synthesis of small and spherical AgNPs while broader SPR bands (two or three) at longer wavelengths are characteristic of anisotropic and large AgNPs [50,51]. So, pH 10 was chosen for the synthesis of *A. alba* and *P. sylvestris* bark extract-derived AgNPs. 

The concentration of AgNO_3_ solution is another parameter that significantly affected the synthesis of AgNPs. As shown in Figure 1C,D, for both *A. alba* and *P. sylvestris* bark extract-derived AgNPs, the absorption increased up to 2 mM AgNO_3_. At 3 mM AgNO_3_, the absorbance started to decrease and the SPR bands became broader and slightly shifted to longer wavelengths. It seems that higher AgNO_3_ concentrations impair the synthesis and moreover, lead to larger AgNPs. The best SPR peak (as intensity, shape, and position) was obtained at 2 mM AgNO_3_, and therefore this concentration was chosen as the optimal one for the synthesis of AgNPs.

The effect of the volume ratio of bark extract to 2 mM AgNO_3_ on AgNPs synthesis was investigated in several proportions (1:9, 2:8, 3:7, 5:5, 7:3, and 9:1). In the case of *A. alba* bark extract, AgNPs were synthesized only when the bark extract and AgNO_3_ were used at a ratio of 1:9 (*v*/*v*); no AgNPs synthesis was observed at other ratios used in the experiment (Figure 1E). For *P. sylvestris* bark extract-derived AgNPs, the highest SPR band was achieved at a similar ratio of bark extract to 2 mM AgNO_3_ (1:9, *v*/*v*); the intensity of the SPR band decreased as the volume of bark extract increased (Figure 1F). Hence, a bark extract to AgNO_3_ (2 mM) ratio of 1:9 (*v*/*v*) was chosen as optimal for the synthesis of AgNPs.

The effect of temperature on AgNPs synthesis was evaluated using temperature values ranging from 30 to 80 °C and previously optimized parameters. In the case of *A. alba* bark extract-derived AgNPs, sharp peaks appeared when the synthesis was conducted at 40, 60, and 80 °C (absorption maximum at 426, 428, and 427 nm, respectively). Smaller and broader peaks were generated at 30, 50, and 70 °C (Figure 1G). For *P. sylvestris* bark extract-derived AgNPs, the best yields were obtained at 30 and 70 °C. At 30 °C, the absorption maximum was observed at 423 nm; at 70 °C, it shifted to 425 nm (Figure 1H). The temperature values of 60, 80, and 70 °C shifted the absorption maximum of *A. alba* and *P. sylvestris* bark extract-derived AgNPs, respectively, to longer wavelengths, indicating that AgNPs size increased. Therefore, 40 and 30 °C were chosen as optimal temperatures for the synthesis of AgNPs from *A. alba* and *P. sylvestris* bark extract, respectively.

To evaluate the effect of reaction time on AgNPs synthesis, *A. alba*/*P. sylvestris* bark aqueous extract was mixed with 2 mM AgNO_3_ (1:9, *v*/*v*, pH of reaction mixture adjusted to 10 with 0.1 N NaOH) and stirred at 40 °C/30 °C for 120 min. AgNPs synthesis was checked at different time intervals (0–120 min) during stirring and at 24 h after the end of stirring. As shown in Figure 1I,J, for both bark extracts, the reduction of Ag^+^ started immediately after the addition of the reducing agent (bark extract) to the AgNO_3_ solution (0 min). At each time interval, an absorbance peak at around 420 nm indicated the synthesis of AgNPs. The yield of AgNPs increased as the reaction time increased. Therefore, a period of 120 min stirring followed by 24 h at rest was considered optimal for the synthesis of AgNPs. 

The optimized experimental parameters for the synthesis of AgNPs were pH 10, the temperature of 30 and 40 °C (for *P. sylvestris* and *A. alba* bark extract-derived AgNPs, respectively), 2 mM AgNO_3_, bark extract to AgNO_3_ (2 mM) ratio of 1:9 (*v*/*v*), and 120 min reaction time (followed by 24 h at rest). 

### 3.3. Characterization of AgNPs

*A. alba* and *P. sylvestris* bark extract-derived AgNPs, synthesized under optimized conditions, were characterized by ATR-FTIR spectroscopy, DLS, TEM, and SEM with EDX analyses.

**Figure 2:**
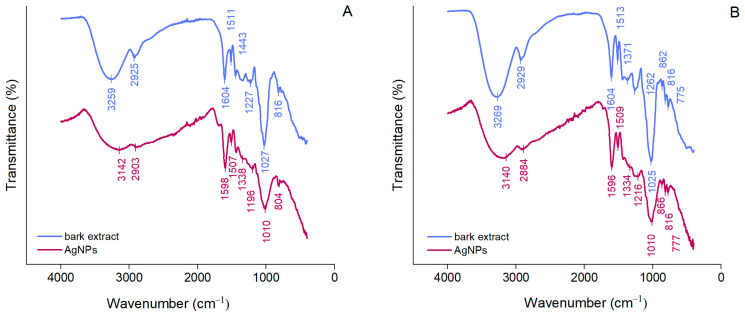
ATR-FTIR spectra of *Abies alba* (**A**) and *Pinus sylvestris* (**B**) bark aqueous extracts and their derived AgNPs.

ATR-FTIR spectroscopy was used to identify the functional groups of the compounds in bark extracts involved in the synthesis and stabilization of AgNPs. As shown in Figure 2, *A. alba* and *P. sylvestris* bark extracts exhibited broad bands at 3259 and 3269 cm^−1^, respectively, corresponding to the stretching vibrations of the hydroxyl groups of polyphenols and polysaccharides [52]. Bands at 2925 and 2929 cm^−1^ (*A. alba* and *P. sylvestris* bark extract, respectively) belong to the stretching vibrations of the methylene groups [53]. Bands around 1600 and 1500 cm^−1^ (both bark extracts), 1443 cm^−1^ (*A. alba* bark extract), and 1262 cm^−1^ (*P. sylvestris* bark extract) could be ascribed to the stretching vibrations of the aromatic rings and =C–O–C groups of flavonoids [29,54]. The bands around 1600 and 1500 cm^−1^ could also belong to the amide region in proteins [29,53,55]. The bands at 1027 and 1025 cm^−1^ (*A. alba* and *P. sylvestris* bark extract, respectively) are characteristic of the C–O–C stretching vibrations in carbohydrates [29,56,57]. The aromatic C–H out-of-plane bending vibrations are indicated by the bands at 816 cm^−1^ (both bark extracts) and 775 cm^−1^ (*P. sylvestris* bark extract), the latter suggesting the presence of flavonoids (B ring) [29,58]. ATR-FTIR spectra of AgNPs illustrate shifts in the bark extract bands confirming the involvement of some phytochemicals (polyphenols, carbohydrates, proteins) in the formation of AgNPs (reduction of Ag^+^ to metallic Ag), but also in their stabilization. The bands at 3259 and 3269 cm^−1^ in *A. alba* and *P. sylvestris* bark extracts, respectively, not only shifted, but they attenuated and broadened, indicating the participation of polyphenols/polysaccharides in the synthesis of AgNPs. 

Polyphenols majorly contributed to the synthesis and stabilization of AgNPs. Their contribution is also supported by a significant decrease in the phenolic contents of the reaction mixtures after the synthesis and separation of AgNPs (supernatants) (*A. alba*: 152.01 ± 11.16 μg/mL in the reaction mixture vs. 18.88 ± 3.09 μg/mL in supernatant; *P. sylvestris*: 608.15 ± 9.33 μg/mL in the reaction mixture vs. 432.71 ± 3.89 μg/mL in the supernatant). 

**Figure 3:**
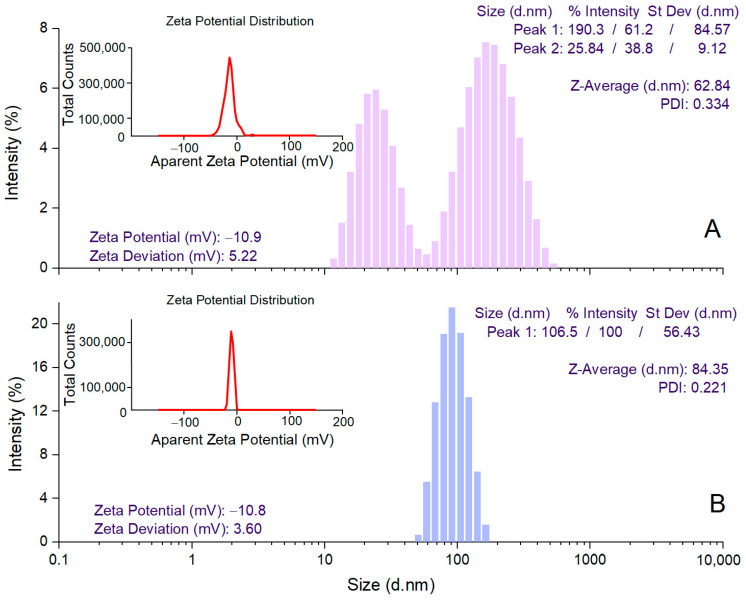
DLS analysis of *Abies alba* (**A**) and *Pinus sylvestris* (**B**) bark extract-derived AgNPs.

DLS analysis was performed to determine the mean hydrodynamic diameter (Z-average), PDI, and zeta potential values for estimating the size of AgNPs in colloidal suspension, their dispersion, and stability, respectively [59]. *A. alba* bark extract-derived AgNPs showed a Z-average value of 62.84 nm and 2 peak values in the hydrodynamic diameter distribution (25.84 and 190.3 nm) (Figure 3A). For *P. sylvestris* bark extract-derived AgNPs, a single peak (106.5 nm) with a narrow base was obtained, indicating better homogeneity; the average particle size (Z-average) was 84.35 nm (Figure 3B). PDI value is an indicator of the homogeneity of the size distribution of AgNPs in colloidal dispersion. Low PDI values (below 0.7) reflect a narrow range of particle sizes and high uniformity [60]. In this study, PDI values indicated that *P. sylvestris* bark extract-derived AgNPs had a narrower distribution and better homogeneity in comparison with those derived from *A. alba* bark extract (0.221 vs. 0.334) (Figure 3A,B). The zeta potential indicates the charge on the surface of AgNPs and predicts their stability in colloidal dispersion, with values of ±10–20 mV reflecting a relatively stable colloid system [55,61]. Both *A. alba* and *P. sylvestris* bark extract-derived AgNPs showed similar negative values for zeta potential (−10.9 and −10.8 mV, respectively) (Figure 3A,B). 

**Figure 4:**
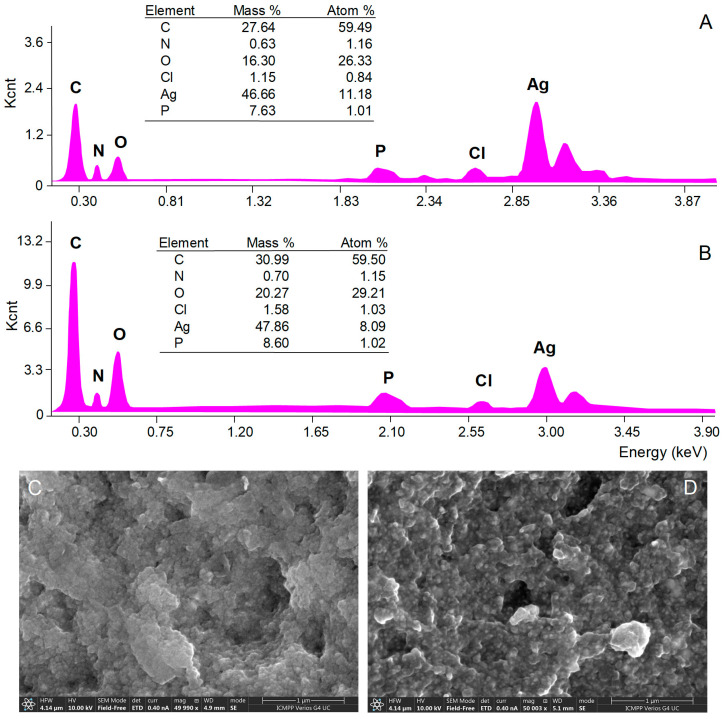
Elemental composition (EDX spectra) and SEM micrographs of *Abies alba* (**A**,**C**) and *Pinus sylvestris* (**B**,**D**) bark extract-derived AgNPs.

The morphology of AgNPs’ surface and their elemental composition were investigated by SEM with EDX analyses. SEM micrograph images showed aggregations of spherical nanoparticles (Figure 4C,D)*,* most probably caused by the procedures used to isolate AgNPs from the reaction mixtures (centrifugation followed by freeze-frying). EDX analysis provides qualitative and quantitative information on the chemical composition of AgNPs [34]. The EDX spectra of both *A. alba* and *P. sylvestris* bark extract-derived AgNPs (Figure 4A,B, respectively) revealed a pronounced signal for metallic Ag at 3 keV which confirmed the formation of AgNPs. Several other signals were registered for C, N, O, P, and Cl belonging to the compounds in bark extracts acting as capping agents [33,62]. 

**Figure 5:**
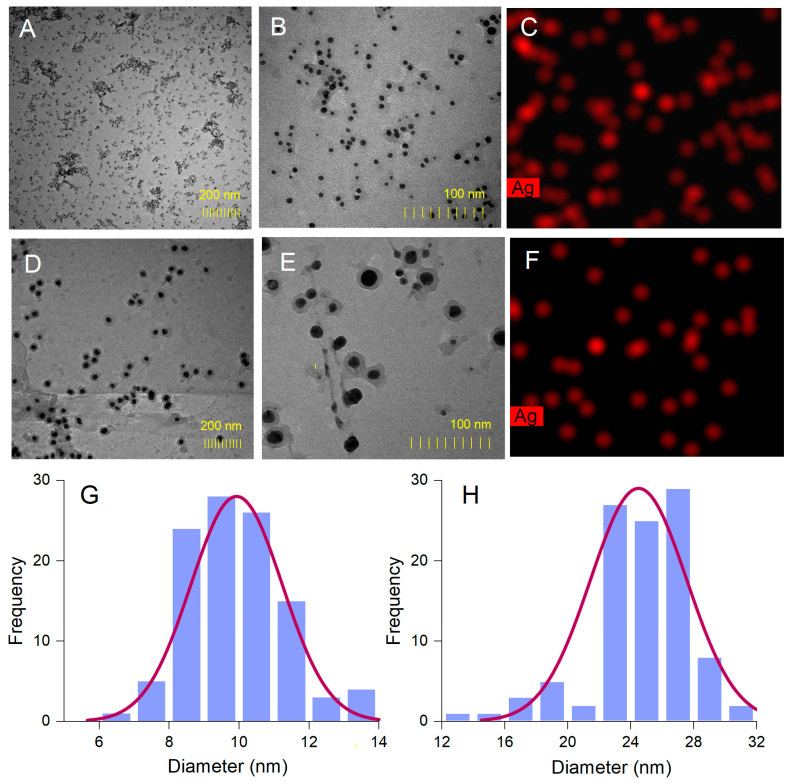
*Abies alba* bark extract-derived AgNPs: TEM images (**A**,**B**), TEM-EDX mapping (**C**), size-distribution histogram (**G**); *Pinus sylvestris* bark extract-derived AgNPs: TEM images (**D**,**E**), TEM-EDX mapping (**F**), and size-distribution histogram (**H**).

The spherical shape of *A. alba* and *P. sylvestris* bark extract-derived AgNPs was confirmed by TEM (Figure 5A,B,D,E). In contrast to SEM micrographs, TEM images showed that the synthesized AgNPs were well-dispersed. This disparity might have its origin in sample preparation for analysis. As already mentioned, AgNPs as pellets, obtained by centrifugation and freeze-drying, were analyzed by SEM, whereas AgNPs as colloidal dispersion were subjected to TEM. Centrifugation followed by freeze-frying promoted AgNPs agglomeration, which was visible in SEM micrographs. Colloidal AgNPs formed in situ had a good dispersion rate, as illustrated by TEM analysis. Both *A. alba* and *P. sylvestris* bark extract-derived AgNPs showed lighter margins attributed to the compounds in bark extracts which adhered to the surface of AgNPs, thus capping and stabilizing them [63,64]. The presence of metallic Ag was revealed by TEM-EDX mapping (Figure 5C,F) confirming the reduction of Ag^+^ to Ag^0^ by the compounds present in bark extracts. *A. alba* bark extract-derived AgNPs ranged from 6 to 13 nm, whereas *P. sylvestris* bark extract-derived AgNPs were larger (12–31 nm). By fitting the histogram data with a Gaussian distribution, the average particle size was found to be 9.92 and 24.49 nm for *A. alba* and *P. sylvestris* bark extract-derived AgNPs, respectively (Figure 5G,H). 

### 3.4. Antioxidant Activity

**Figure 6:**
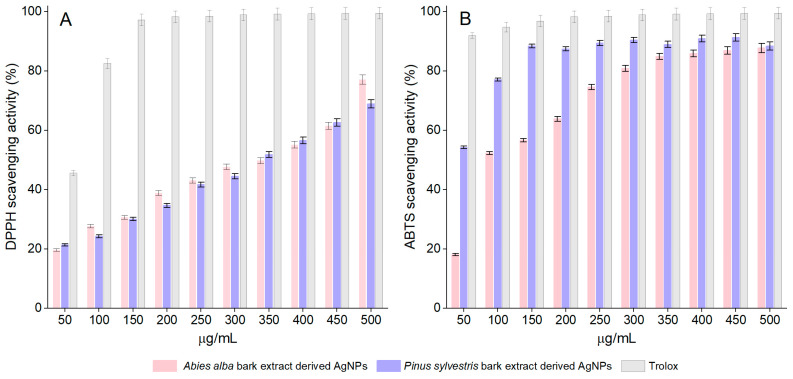
DPPH scavenging activity (**A**) and ABTS scavenging activity (**B**) of *Abies alba* and *Pinus sylvestris* bark extract-derived AgNPs and Trolox; significant differences (*p* < 0.001) between *Abies alba*/*Pinus sylvestris* bark extract-derived AgNPs and Trolox (at all tested concentrations).

Both *A. alba* and *P. sylvestris* bark extract-derived AgNPs scavenged the DPPH radical in a dose-dependent manner. For *A. alba* bark extract-derived AgNPs, the scavenging activity increased from 19.65 ± 1.24% (at 50 μg/mL) to 77.13 ± 0.40% (at 500 μg/mL). In the same concentration range, the scavenging ability of *P. sylvestris* bark extract-derived AgNPs varied from 21.42 ± 0.53% to 69.00 ± 1.20%. At 50 μg/mL, Trolox, the positive control, showed 45.62 ± 0.56% scavenging activity, whereas at 150 μg/mL, it almost completely scavenged the DPPH radical (97.20 ± 0.94%) (Figure 6A). For EC_50_ calculation, additional dilutions of Trolox up to 10 μg/mL (13.48 ± 0.65% DPPH scavenging activity) were tested. According to the EC_50_ values, *P. sylvestris* bark extract-derived AgNPs were slightly more active than *A. alba* bark extract-derived AgNPs; Trolox was approximately six times more active than bark extract-derived AgNPs (Table 1). 

The synthesized AgNPs dose-dependently scavenged the ABTS radical cation. The scavenging activity of *A. alba* and *P. sylvestris* bark extract-derived AgNPs increased from 18.15 ± 0.36% and 54.26 ± 1.08%, respectively (at 50 μg/mL) to 87.76 ± 1.75% and 88.45 ± 1.76%, respectively (at 500 μg/mL). At 50 μg/mL, Trolox showed 91.98 ± 1.83% scavenging activity, whereas at 150 μg/mL, its scavenging activity increased to 99.50 ± 1.99% (Figure 6B). The ABTS radical cation scavenging activity varied as follows: Trolox ˃ *P. sylvestris* bark extract-derived AgNPs ˃ *A. alba* bark extract-derived AgNPs (Table 1).

### 3.5. Cytotoxic Activity

**Figure 7:**
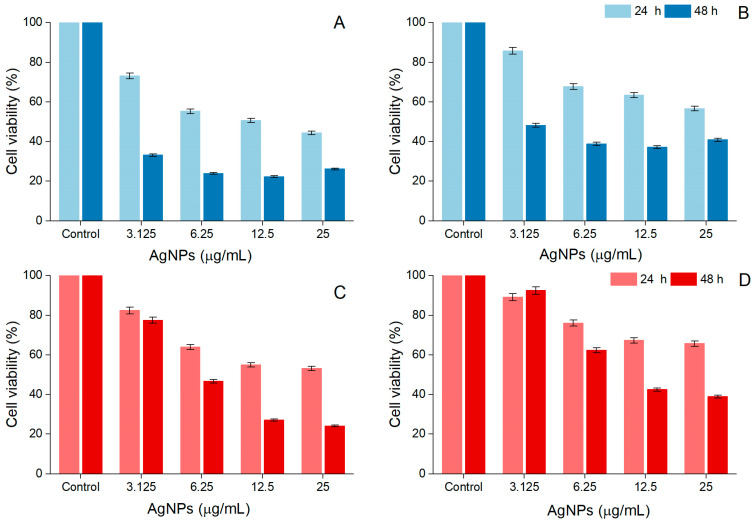
Viability of A-375 human malignant melanoma cells exposed to *Abies alba* (**A**) and *Pinus sylvestris* bark extract-derived AgNPs (**C**); viability of African green monkey kidney (Vero) cells exposed to *Abies alba* (**B**) and *Pinus sylvestris* bark extract-derived AgNPs (**D**); significant differences (*p* < 0.001) between *Abies alba*/*Pinus sylvestris* bark extract-derived AgNPs (at all tested concentrations) and control.

*A. alba* and *P. sylvestris* bark extract-derived AgNPs negatively impacted cell viability in a time- and dose-dependent manner. After 48 h treatment, the viability of A-375 cells exposed to *A. alba* bark extract-derived AgNPs (12.5 μg/mL) significantly decreased to 22.32 ± 3.48%. At higher concentrations (25 μg/mL), a slight increase in cell viability (26.12 ± 0.57%) was noticed (Figure 7A). A similar tendency was observed in Vero cells, namely 37.11 ± 1.84% and 40.90 ± 2.65% viability in cells treated with 12.5 and 25 μg/mL *A. alba* bark extract-derived AgNPs, respectively (Figure 7B). When A-375 and Vero cells were exposed to *P. sylvestris* bark extract-derived AgNPs (12.5 and 25 μg/mL) for 48 h, the following cell viability percentages were detected: 27.02 ± 0.36%, 24.10 ± 0.72%, and 42.46 ± 1.20%, 38.95 ± 3.15%, respectively (Figure 7C,D). According to the IC_50_ values (after 48 h treatment), *A. alba* bark extract-derived AgNPs were more cytotoxic to A-375 cells; both bark extract-derived AgNPs were less cytotoxic to Vero cells (Table 1). 

**Figure 8:**
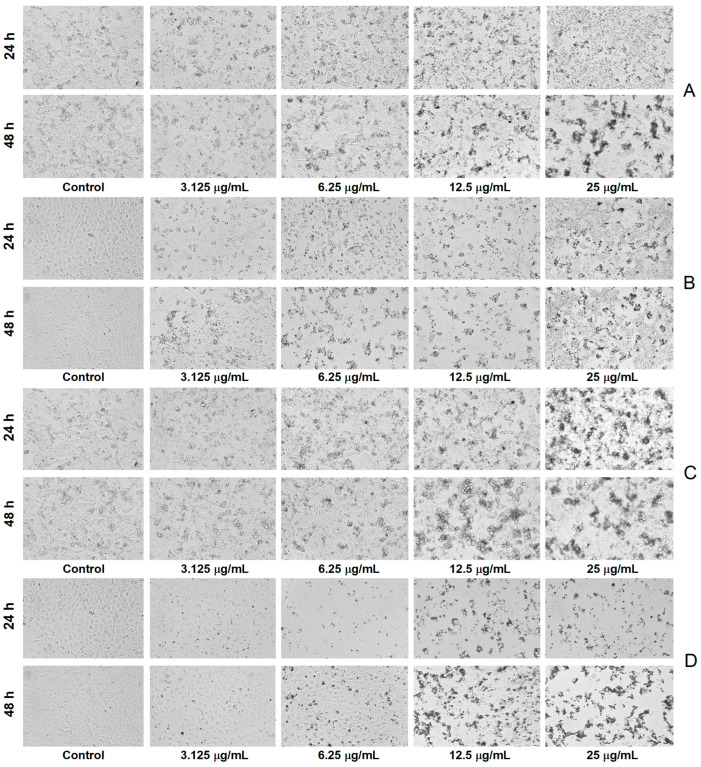
Bright-field morphological aspects of cells exposed to *Abies alba* bark extract-derived AgNPs ((**A**)—A-375 human malignant melanoma cells, (**B**)—African green monkey kidney (Vero) cells) and *Pinus sylvestris* bark extract-derived AgNPs ((**C**)—A-375 human malignant melanoma cells, (**D**)—African green monkey kidney (Vero) cells); scale bar 100 µm (10× magnification).

The microscopic investigation revealed a decrease in cell density and substrate adhesion capacity together with membrane shrinkage after exposure to *A. alba* and *P. sylvestris* bark extract-derived AgNPs, the processes being more intense after 48 h exposure. In contrast, the control cells showed a high confluence of the monolayer, intact cell membrane, and smooth morphology (without roughness) (Figure 8). 

### 3.6. Antibacterial Activity

The antibacterial activity of AgNPs was investigated against five pathogenic reference strains, including two resistant ones: methicillin-resistant *S. aureus* (MRSA) ATCC 43300 and *S. epidermidis* ATCC 12228. The former also showed resistance to oxacillin and gentamicin, whereas the latter proved to be resistant to tetracycline [65]. According to the MIC values (Table 2), *A. alba* bark extract-derived AgNPs possessed good antibacterial activity, being more active than *P. sylvestris* bark extract-derived AgNPs against all tested bacteria except *P. aeruginosa* ATCC 9027. In addition, *A. alba* bark extract-derived AgNPs inhibited the growth of resistant bacteria (MRSA ATCC 43300, *S. epidermidis* ATCC 12228) while *P. sylvestris* bark extract-derived AgNPs showed no activity against these two strains in the tested concentration range (0.24–250 μg/mL). 

## 4. Discussion

The use of plant extracts for the preparation of AgNPs has important advantages over other methods (physical, chemical, mechanical, and microbial). Plant extract-mediated AgNPs synthesis is simple, fast, and cost-effective. Phytochemicals in plant extracts play a dual role, serving as both reducing agents and stabilizers of AgNPs. In addition, the method is eco-friendly as it produces less environmental contamination [8]. To date, different parts of coniferous trees (bark, stem, needles, cones, and even exudates such as gum) have been used for the synthesis of AgNPs with various biological effects (antimicrobial, anticancerous, antioxidant, and antipyretic) [19]. 

In our study, AgNPs were prepared using *A. alba* and *P. sylvestris* bark aqueous extracts. Both extracts were initially subjected to LC-HRMS/MS resulting in the identification/tentative identification of 22 compounds belonging to various phytochemical classes [25,30,66,67,68,69,70,71,72,73,74,75]. Of these compounds, several phenolics have already been reported in both conifer barks: catechin, gallocatechin, epicatechin, and lariciresinol in *A. alba* bark [23,76] and *O*-hexosides (*O*-glucosides) of hydroxybenzoic acid, vanillic acid, and taxifolin, procyanidin dimer, catechin, and taxifolin in *P. sylvestris* bark [21,25,27]. Apart from these phenolics, our study also spotted coumaroylquinic acids, terpenoid glycosides, and organic acids. Coumaroylquinic acids have been previously identified in the bark of other *Pinus* species such as *Pinus nigra* [29] and *Pinus densiflora* [77]. Mentha-2,8-dien-1-ol, menthane-3,8-diol, menth-2-en-1-ol, and cymen-8-ol are volatile monoterpenoids present in the essential oils isolated from various coniferous species [78,79,80,81,82,83]. On the basis of mass spectral data (Supplementary Table S1), previous investigations on MS/MS fragmentation of monoterpene glycosides [84,85], and the KNApSacK database [71], seven glycosides of the previously mentioned volatile monoterpenoids were tentatively identified in both bark extracts. Most fragment ions observed in the MS/MS spectra of these glycosides (compounds **9**, **18**, **22–26**) resulted from the loss of the hexosyl (162 Da), deoxyhexosyl (146 Da), and pentosyl (132 Da) residues, deprotonation, dehydration, and internal cleavage of sugar moieties. In addition, two dicarboxylic acids, azelaic (1,9-nonanedionic acid, compound **21**) and sebacic (1,10-decadienoic acid, compound **27**) acids, were tentatively identified. The former is an important signaling molecule in systemic acquired resistance, an inducible defense mechanism providing protection against pathogen infection [86]. Among the phytochemicals identified in bark extracts, polyphenols are highly involved in the synthesis and stabilization of AgNPs. The phenolic hydroxyl groups are responsible for the conversion of Ag^+^ to Ag^0^. At the same time, they cap around AgNPs, thus stabilizing them [3,8]. The involvement of polyphenols in the reduction of Ag^+^ and stabilization of *A. alba* and *P. sylvestris* bark extract-derived AgNPs is supported by ATR-FTIR data and also by a reduction of the total phenolic content in reaction mixtures after the synthesis and separation of AgNPs.

The biological effects of plant-derived AgNPs depend not only on the components of plant extracts but also on their physicochemical properties which are influenced by the synthesis parameters (pH, AgNO_3_ concentration, ratio between the volumes of plant extract, AgNO_3_, temperature, and reaction time) [3,12,33,35]. Therefore, in our study, we optimized the reaction conditions, taking into consideration the aforementioned parameters and monitoring the synthesis of AgNPs by UV-Vis spectroscopy. pH significantly affects the synthesis and stability of AgNPs. An alkaline pH facilitates the deprotonation of phenolic hydroxyl groups, thus enhancing their reduction abilities and consequently the synthesis of AgNPs [35]. At the same time, the amount of negative charge on the surface of AgNPs increases, the electrostatic repulsion between AgNPs becomes stronger, and their aggregation is lowered, resulting in small-sized, well-dispersed, and stable AgNPs. In addition, AgNPs synthesized in alkaline conditions have more regular shapes, usually spherical and hexagonal [87]. Basic pH values (8–12) were found to be optimal for the synthesis of AgNPs using extracts prepared from *Picea abies* bark [88,89], *Pinus eldarica* bark [90], *Pinus thunbergii* cones [91], *Bryophyllum pinnatum* leaves [33], *Oryza sativa* (Thai pigmented rice, riceberry) [35], *Syzygium jambos* leaves and bark [92], and *Givotia moluccana* leaves [53]. On the other hand, there are also studies reporting an optimal synthesis of AgNPs at neutral pH [51]. In our study, the synthesis of AgNPs was supported by an alkaline medium (pH 10). Another factor which dramatically affects the synthesis and stability of AgNPs is AgNO_3_ concentration. Its increase results in higher yields of AgNPs but also a higher agglomeration rate. Moreover, an increase in AgNO_3_ concentration above 10 mM causes the deposition of AgNO_3_ on the surface of AgNPs, which may enhance their toxicity. Most studies showed that 1 mM AgNO_3_ is the optimal concentration for AgNPs synthesis [51]. However, in some cases, small-sized AgNPs were synthesized using higher AgNO_3_ concentrations. AgNPs of 15 and 20–50 nm were synthesized using 2 mM AgNO_3_ and extracts obtained from *Crocus sativus* wastages [93] and *Chrysanthemum morifolium* flowers [94], respectively. AgNPs of 30–50 nm were obtained from *Chenopodium murale* leaf extract and 5 mM AgNO_3_ [95]. The synthesis of monodispersed, spherical, and 9 nm sized AgNPs was achieved using *Hibiscus cannabinus* leaf extract and 5 mM AgNO_3_ [96]. In our study, 9.92 and 24.49 nm sized AgNPs (according to TEM data) were produced from *A. alba* and *P. sylvestris* bark aqueous extracts, respectively, and 2 mM AgNO_3_ in a ratio of 1:9 (*v*/*v*); an increase in the bark extract volume (ratios of 2:8, 3:7, 5:5, 7:3, and 9:1) impaired the synthesis of AgNPs. Other studies reported an optimal synthesis of AgNPs using *Picea abies* bark extract [89], *Aloe vera* leaf extract [97], *Rubus glaucus* fruit extract [98], *Thymus kotschyanus* plant extract [99], *Juglans regia* seed extract [100] or *Prunus serotina* cherry extract [101] and AgNO_3_ (1 mM) in a ratio of 1:10 (*v*/*v*). An increase in AgNO_3_ volume (*P. abies* bark extract and AgNO_3_ in ratios of 1:50 and 1:100) significantly reduced the accumulation of AgNPs [89]. Temperature plays an important role in the synthesis of AgNPs. An increase in temperature enhances the kinetic energy of molecules and accelerates the reaction rate resulting in smaller-sized AgNPs and less AgNPs agglomeration [102]. On the other hand, high kinetic energy might enable multiple collisions and consequently, the aggregation and growth of AgNPs [103]. In our study, the optimal temperatures for the synthesis of *P. sylvestris* and *A. alba* bark extract-derived AgNPs were 30 and 40 °C, respectively. According to the literature data, a high-yield synthesis of uniformly sized, small, and spherical AgNPs was achieved at temperatures of 30–40 °C using leaf extracts of *Hibiscus cannabinus* [96], aloe [103], *Cynodon dactylon* [104], *Ocimum sanctum* [105], *Tephrosia purpurea* [106], *O. tenuiflorum*, *Solanum tricobatum, Syzygium cumini*, and *Centella asiatica* and peel extract of *Citrus sinensis* [107]. Reaction time affects both the yield and stability of AgNPs. Prolonged reaction times increase the yield of AgNPs but promote agglomeration of AgNPs, decreasing their stability [108]. There have been reported various reaction times in plant-mediated synthesis of AgNPs, e.g., 20 min [103], 30 min [35], 45 min [102], 1 h [104], 24–48 h [107], and 72 h [34]. In our study, a reaction time of 120 min (followed by 24 h at rest) was found to be optimal for the synthesis of AgNPs using *A. alba* and *P. sylvestris* bark extracts. 

The optimized parameters (pH 10, 30, and 40 °C for *P. sylvestris* and *A. alba* bark extract-derived AgNPs, respectively, 2 mM AgNO_3_, bark extract to 2 mM AgNO_3_ ratio of 1:9, *v*/*v*, and 120 min reaction time, followed by 24 h at rest) led to the synthesis of well-dispersed, spherical, small, and relatively stable AgNPs. Both DLS and TEM determined smaller average particle sizes for *A. alba* bark extract-derived AgNPs (62.84 and 9.92 nm, respectively, vs. 84.35 and 24.49 nm, respectively, for *P. sylvestris* bark extract-derived AgNPs). The difference in the average size between *A. alba* and *P. sylvestris* bark extract-derived AgNPs could be attributed to the different temperature values used in AgNPs synthesis (40 °C for *A. alba* bark extract-derived AgNPs, 30 °C for *P. sylvestris* bark extract-derived AgNPs) and AgNPs outer shell consisting of capping/stabilizing compounds. According to Verma and Mehata [102], as the temperature increases, the reaction rate quickens, resulting in smaller-sized AgNPs. In contrast to TEM, DLS analysis determined larger sizes (62.84 and 84.35 nm for *A. alba* and *P. sylvestris* bark extract-derived AgNPs, respectively). The discrepancy might be ascribed to the fact that DLS estimates the hydrodynamic diameter of AgNPs (the metallic core of AgNPs with the compounds adsorbed on their surface and the solvation shell), whereas TEM measures the geometric size of AgNPs [109]. The synthesized AgNPs were negatively charged (zeta potential values of −10.9 and −10.8 mV for *A. alba* and *P. sylvestris* bark extract-derived AgNPs, respectively). As already mentioned, polyphenols were involved in the synthesis and stabilization of AgNPs. According to Yang and Li (2013), an alkaline pH enhances the deprotonation of the phenolic hydroxyl groups of capping AgNPs, resulting in negatively charged hydroxylate ions on the surface of AgNPs [110]. The electrostatic repulsive forces between negatively charged AgNPs prevent their agglomeration, resulting in high dispersity and stability [55,111].

The synthesized AgNPs showed antioxidant, anticancer, and antibacterial effects. Both *A. alba* and *P. sylvestris* bark extract-derived AgNPs scavenged the DPPH radical (EC_50_ = 351.70 and 336.70 μg/mL, respectively) and ABTS radical cation (EC_50_ = 98.60 and 37.80 μg/mL, respectively), the scavenging activity being attributable to elemental Ag [112] but also to phytochemicals covering the surface of AgNPs [101,113]. The differences in antioxidant potency between *A. alba* and *P. sylvestris* bark extract-derived AgNPs are owing to the capping/stabilizing phytochemicals [38,101]. Our results are consistent with previous studies reporting DPPH [33,37,95,98,99,101,112,114] and ABTS [33,38,115,116,117] scavenging potential for AgNPs capped with plant constituents. In a similar DPPH experimental protocol, AgNPs synthesized using *Elephantopus scaber* leaf extract exhibited an IC_50_ value of 126.6 μg/mL [37]. Other studies used various experimental protocols to evaluate the DPPH scavenging activity of the phytofunctionalized AgNPs. AgNPs synthesized using *Bryophyllum pinnatum* leaf extract scavenged the DPPH free radical with an IC_50_ value of 89.05 μg/mL [33]. At 500 μg/mL, AgNPs prepared using *Iresine herbstii* leaf extract inactivated the radical by more than 70% [112]. At 100 μg/mL, the DPPH scavenging activity of AgNPs synthesized from *Rhododendron dauricum* flower extract was comparable to that of Trolox (approx. 70%) [114]. In various ABTS experimental protocols, AgNPs synthesized using extracts of *Hypericum perforatum* (aerial parts), *Origanum onites* (aerial parts), *Argyreia nervosa* (leaves), and *Bryophyllum pinnatum* (leaves) showed IC_50_ values of 26.78 [38], 5.16 [115], 44.3 [116], and 259.14 μg/mL, respectively [33]. 

The anticancer potential of AgNPs has recently gained much attention. Various mechanisms are involved in the anticancer activity of AgNPs such as alteration of cell/lysosomal membrane integrity, increase in oxidative stress, induction of apoptosis, cell cycle arrest, and inhibition of angiogenesis [48,118]. The anticancer activity of AgNPs is considerably influenced by their size, shape, and zeta potential. A smaller size, an irregular shape, and a negative zeta potential are essential for good anticancer activity [119]. The use of plant extracts for the synthesis of AgNPs has produced phytofunctionalized AgNPs with remarkable anticancer properties. Plant extract-derived AgNPs proved to be active against different human cancer cell lines (lung, breast, liver, cervical, prostate, colon, ovarian, etc.); some phytofunctionalized AgNPs displayed low IC_50_ values (<100 μg/mL) and/or showed less cytotoxicity against normal cells [8,120,121]. To the best of our knowledge, the cytotoxic potential of phytofunctionalized AgNPs against human melanoma cells has been scarcely investigated [122]. González-Pedroza et al. [123] have recently studied the cytotoxic effects of AgNPs synthesized using *Annona muricata* leaf and fruit peel aqueous extracts on A-375 human malignant melanoma cells and found a remarkable activity (IC_50_ = 2.004 and 1.285 μg/mL, respectively), with less toxicity to normal macrophages (IC_50_ = 10.7 and 13.7 μg/mL, respectively). *A. muricata* aqueous extracts contain polyphenols, acetogenins, and citric acid, which are responsible not only for the synthesis and stabilization of AgNPs but also for their anticancer activity. Acetogenins, for example, are well known for their pro-apoptotic effects [123]. Another study reported a significant reduction in the viability of A-375 cells after exposure to AgNPs obtained from the homeopathic mother tinctures (ethanolic extracts, 70% ethanol, *v*/*v*) of *Gelsemium sempervirens*, *Thuja occidentalis*, *Phytolacca decandra*, and *Hydrastis canadensis* (IC_50_ = 80, 120, 78, and 100 μg/mL, respectively). All AgNPs showed pro-apoptotic effects associated with DNA fragmentation, cell cycle arrest at the G2/M phase, and caspase-3 activation [119]. AgNPs synthesized using boswellic acid (isolated from the gum resin of *Boswellia serrata*) exerted cytotoxic activity on G361 human malignant melanoma cells, and the effect is comparable to that of doxorubicin [124]. In our study, *A. alba* and *P. sylvestris* bark extract-derived AgNPs showed significant cytotoxicity toward A-375 human malignant melanoma cells (IC_50_ = 2.40 and 6.02 μg/mL, respectively), being less cytotoxic against normal Vero cells (IC_50_ = 2.76 and 9.71 μg/mL, respectively) (after 48 h treatment). The smaller-sized *A. alba* bark extract-derived AgNPs exhibited higher cytotoxicity against both cell lines. Smaller AgNPs are more cytotoxic due to their more efficient interaction with cell membranes, resulting in a higher penetration rate and cellular uptake and consequently higher antitumor efficacy [3,13,121,125]. In addition, phytochemical capping AgNPs undoubtedly contribute to the anticancer activity of phytofunctionalized AgNPs. Our study showed that polyphenols in *A. alba* and *P. sylvestris* bark extracts are involved in the synthesis and stabilization of AgNPs. Among these polyphenols, catechins (catechin, epicatechin, gallocatechin, procyanidin oligomers) have been reported to inhibit melanoma cell growth and invasion by multiple mechanisms (cell cycle arrest, decrease in cyclooxygenase-2 expression, prostaglandin E_2_ production, ERK1/2 phosphorylation, and NFκBp65 activation) [126]. Moreover, a pine bark extract—*Pinus maritima* (syn. *Pinus pinaster*), having 61.26 mg catechin/g, induced apoptosis in A-375 cells by increasing caspase-3 expression and activity. In addition, the extract reduced oxidative stress and down-regulated matrix metallopeptidase-9, an enzyme responsible for cell invasion and metastasis [127]. *A. alba* and *P. sylvestris* bark extract-derived AgNPs also altered cell morphology, most probably due to cytoskeleton damage [123].

AgNPs are well-known antimicrobial agents being active against Gram-positive and Gram-negative bacteria, fungi, and viruses, including drug-resistant strains. In brief, AgNPs adhere to the microbial cell surface through electrostatic forces disrupting the cell membrane integrity, penetrating inside the cell, and interacting with proteins, lipids, ribosomes, and DNA, causing severe cellular dysfunctions. AgNPs increase cellular oxidative stress and alter important signal transduction pathways (tyrosine phosphorylation) causing cell damage and death [4]. The antibacterial potential of AgNPs can be significantly improved by using plant extracts for their synthesis. Extracts from different parts of plants (leaves, flowers, fruits, seeds, peels, roots, rhizomes, bark, stem, and even gum, latex, and callus) and whole plants have been used for the synthesis of AgNPs with antibacterial effects. The capping phytochemicals but also the size of AgNPs significantly impact the antibacterial activity. A small size (<50 nm) provides a greater surface of interaction with the bacterial membrane, which enhances the antibacterial potency [12,128]. In our study, the stronger antibacterial activity of *A. alba* bark extract-derived AgNPs can be attributed, in large part, to their smaller size (9.92 vs. 24.49 nm for *P. sylvestris* bark extract-derived AgNPs). In addition, *A. alba* bark extract-derived AgNPs displayed the lowest MIC values (18.75 μg/mL) against two Gram-positive bacteria (*S. aureus* ATCC 25923 and *S. epidermidis* ATCC 12228) which supports the contribution of capping phytochemicals to the antibacterial activity of AgNPs. As already mentioned, “naked” AgNPs are more effective against Gram-negative bacteria, whereas the phytofunctionalized ones have a broader antibacterial spectrum, including Gram-positive bacteria. Polyphenols, which cap and stabilize *A. alba* and *P. sylvestris* bark extract-derived AgNPs, have antimicrobial properties [31]. Catechins, for example, are more effective against Gram-positive bacteria. The antibacterial activity of catechins is associated with cytoplasmic membrane damage, alteration of membrane integrity, and cellular leakage. Catechins easily bind to the peptidoglycan layer of Gram-positive bacteria but hardly penetrate the lipopolysaccharide layers of Gram-negative bacteria [129]. AgNPs synthesized using plant extracts demonstrated efficacy against various bacteria, including those tested in the present study. *Eucalyptus globulus* leaf extract-derived AgNPs displayed antibacterial activity against MSSA, MRSA, and extended-spectrum β-lactamase *E. coli* and *P. aeruginosa* clinical isolates with MIC values of 30, 27, 36, and 27 μg/mL, respectively [130]. The MIC values of AgNPs prepared using *Musa paradisiaca* peel extract against MSSA (*S. aureus* ATCC 6538), *E. coli* ATCC 8739, and *P. aeruginosa* ATCC 9027 were 5.1, 3.4, and 1.7 μg/mL, respectively [34]. AgNPs obtained from *Alpinia katsumadai* seed extract inhibited the growth of *E. coli* (MIC = 20 μg/mL) and *P. aeruginosa* (MIC = 40 μg/mL) [131].

## 5. Conclusions

The results of the present study clearly demonstrate that *Abies alba* and *Pinus sylvestris* bark can be successfully used for the synthesis of bioactive AgNPs. *Abies alba* and *Pinus sylvestris* bark extract-derived AgNPs were well-dispersed, spherical, and small-sized (9.92 and 24.49 nm, respectively). The synthesized AgNPs had a pronounced cytotoxic activity against A-375 human malignant melanoma cells, being less cytotoxic to normal cells. They were also found to have antioxidant activity and antibacterial effects against Gram-positive and Gram-negative bacteria. *Abies alba* bark extract-derived AgNPs demonstrated stronger toxicity against A-375 human malignant melanoma cells as well as stronger antibacterial activity against *Staphylococcus aureus* (methicillin-sensitive and methicillin-resistant), *Staphylococcus epidermidis*, and *Escherichia coli*. The bioactivity of synthesized AgNPs was influenced by their size and capping/stabilizing phytochemicals on their surface. In conclusion, *Abies alba* and *Pinus sylvestris* bark extract-derived AgNPs exhibited antioxidant, antibacterial, and cytotoxic effects against A-375 human malignant melanoma cells. Further studies are needed for an in-depth assessment of their antioxidant, anticancer, and antimicrobial potential and also biocompatibility by using more normal tumor cell lines and bacterial strains.

## Figures and Tables

**Table 1:** Antioxidant and cytotoxic activity of *Abies alba* and *Pinus sylvestris* bark extract-derived AgNPs.

Sample	EC_50_ (μg/mL)		IC_50_ (μg/mL) *	
	Antioxidant Activity		Cytotoxicity (MTT Assay)	
	DPPH Assay	ABTS Assay	A-375 Cells	Vero Cells
*A. alba* bark extract-derived AgNPs	351.7 ± 3.5	98.6 ± 0.3	2.40 ± 0.21	2.76 ± 0.39
*P. sylvestris* bark extract-derived AgNPs	336.7 ± 3.7	37.8 ± 0.4	6.02 ± 0.61	9.71 ± 0.87
Trolox	56.2 ± 0.8	11.4 ± 0.1	ND	ND

ND, not determined; * after 48 h treatment.

**Table 2:** Antibacterial activity of *Abies alba* and *Pinus sylvestris* bark extract-derived AgNPs.

Sample	MIC (μg/mL)				
	*S. aureus* ATCC 25923	*S. aureus* ATCC 43300 (MRSA)	*S. epidermidis* ATCC 12228	*E. coli* ATCC 25922	*P. aeruginosa* ATCC 9027
*A. alba* bark extract-derived AgNPs	18.75	75.00	18.75	37.50	75.00
*P. sylvestris* bark extract-derived AgNPs	75.00	NA	NA	75.00	75.00
Ciprofloxacin	2.00	1.00	0.12	0.03	0.50

NA, no activity.

## Data Availability

The data presented in this study are available upon request from the corresponding author (Anca Miron, anca.miron@umfiasi.ro).

## Supplementary Materials

The following supporting information can be downloaded at: https://www.mdpi.com/article/10.3390/antiox12040797/s1, Figure S1: Base peak chromatograms of *Abies alba* and *Pinus sylvestris* bark aqueous extracts; Table S1: Compounds identified/tentatively identified in *Abies alba* and *Pinus sylvestris* bark aqueous extracts. See Refs. [25,30,66,67,68,69,70,71,72,73,74,75].
