# Supplementary material for: Silver Nanoparticles Synthesized from Abies alba and Pinus sylvestris Bark Extracts: Characterization, Antioxidant, Cytotoxic, and Antibacterial Effects
Source: Antioxidants (Basel). 2023 Mar 24;12(4):797. doi: 10.3390/antiox12040797 (PMC10135277; doi:10.3390/antiox12040797)
Supplement: Supplementary file 1 [file antioxidants-12-00797-s001.zip › antioxidants-2225902-SI.docx]

Supplementary Materials to Article

Silver Nanoparticles Synthesized from *Abies alba* and *Pinus sylvestris* Bark Extracts: Characterization, Antioxidant, Cytotoxic, and Antibacterial Effects

Irina Macovei ^1^, Simon Vlad Luca ^2^, Krystyna Skalicka-Woźniak ^3^, Cristina Elena Horhogea ^4,^*, Cristina Mihaela Rimbu ^4^, Liviu Sacarescu ^5^, Gabriela Vochita ^6^, Daniela Gherghel ^6^, Bianca Laura Ivanescu ^1^, Alina Diana Panainte ^1^, Constantin Nechita ^7^, Andreia Corciova ^1^ and Anca Miron ^1,^*

1 Faculty of Pharmacy, Grigore T. Popa University of Medicine and Pharmacy, 700115 Iasi, Romania; [irina-macovei@umfiasi.ro](mailto:irina-macovei@umfiasi.ro) (I.M.); [bianca.ivanescu@umfiasi.ro](mailto:bianca.ivanescu@umfiasi.ro) (B.L.I.); [alina.gudruman@umfiasi.ro](mailto:alina.gudruman@umfiasi.ro) (A.D.P.); [maria.corciova@umfiasi.ro](mailto:maria.corciova@umfiasi.ro) (A.C.); [anca.miron@umfiasi.ro](mailto:anca.miron@umfiasi.ro) (A.M.)

2 Biothermodynamics, TUM School of Life Sciences, Technical University of Munich, D-85354 Freising, Germany; [vlad.luca@tum.de](mailto:vlad.luca@tum.de)

3 Department of Natural Products Chemistry, Medical University of Lublin, Lublin 20-093, Poland; [kskalicka@pharmacognosy.org](mailto:kskalicka@pharmacognosy.org) (K.S.W.)

4 Department of Public Health, Ion Ionescu de la Brad University of Life Sciences, 700489 Iasi, Romania; [chorhogea@uaiasi.ro](mailto:chorhogea@uaiasi.ro) (C.E.H.); [crimbu@yahoo.com](mailto:crimbu@yahoo.com) (C.M.R.)

5 Petru Poni Institute of Macromolecular Chemistry, 700487 Iasi, Romania; [livius@icmpp.ro](mailto:livius@icmpp.ro) (L.S.)

6 NIRDBS, Institute of Biological Research Iasi, 700107 Iasi, Romania; [gabriela.vochita@icbiasi.ro](mailto:gabriela.vochita@icbiasi.ro) (G.V.); [daniela.gherghel@icbiasi.ro](mailto:daniela.gherghel@icbiasi.ro) (D.G.)

7 Marin Dracea National Institute for Research and Development in Forestry, Campulung Moldovenesc 725100, Romania; [ncincds@gmail.com](mailto:ncincds@gmail.com) (C.N.)

* Correspondence: [chorhogea@uaiasi.ro](mailto:chorhogea@uiasi.ro) (C.E.H.); [anca.miron@umfiasi.ro](mailto:anca.miron@umfiasi.ro) (A.M.)

**S1. Chemical Characterization of Bark Extracts**

*A. alba* and *P. sylvestris* bark aqueous extracts were screened by LC-HRMS/MS. Twenty-two compounds were tentatively identified by comparing their mass spectral data with those found in literature reports and KNApSacK database; the identity of four compounds was confirmed with standards (Figure S1, Table S1). Quantitative studies showed higher phenolic content in *P. sylvestris* bark extract (6081.53 ± 93.31 vs. 1520.12 ± 111.63 μg/mL in *A. alba* bark extract).

**
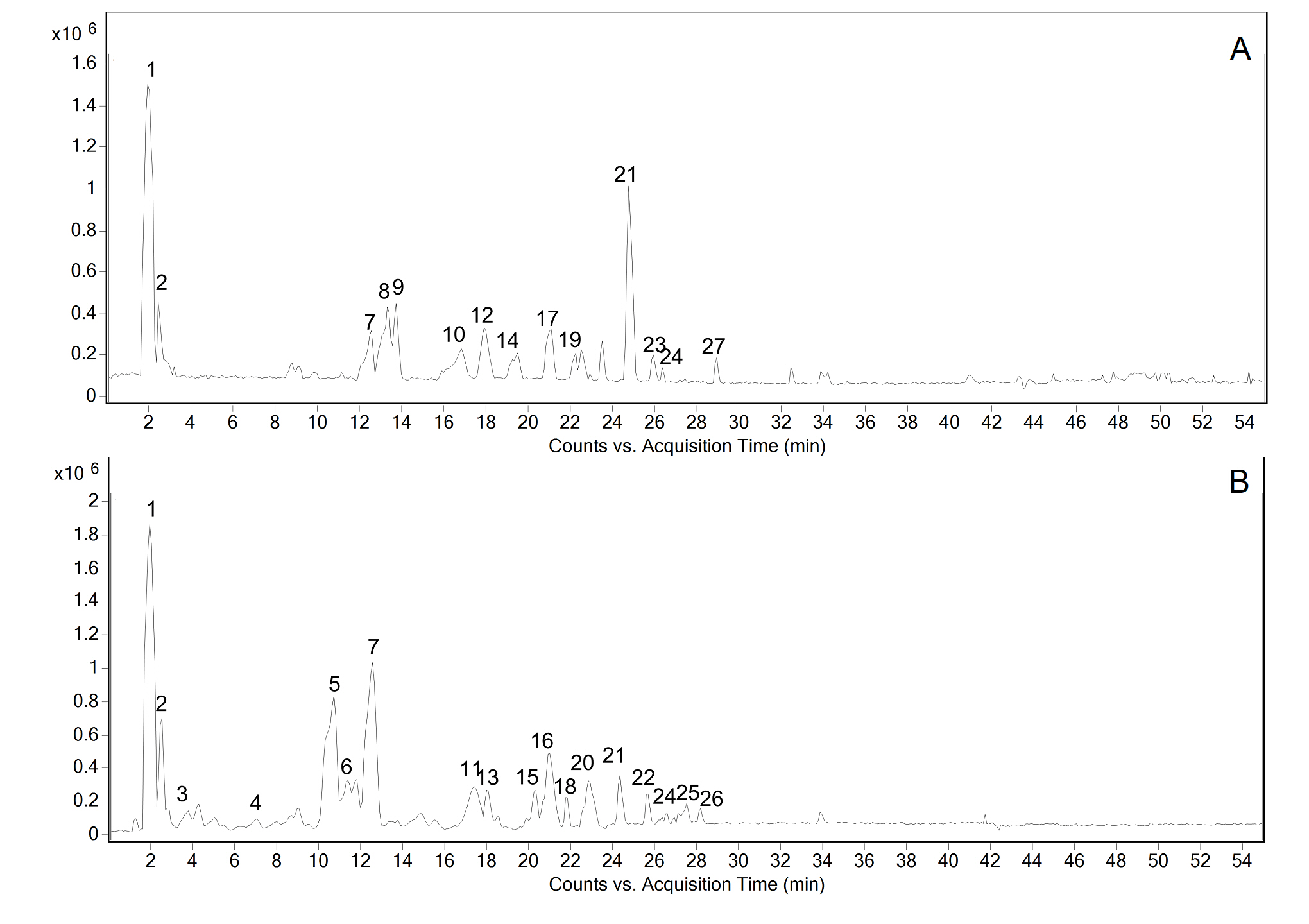
**

**Figure S1.** Base peak chromatograms of *Abies alba* (**A**) and *Pinus sylvestris* (**B**) bark aqueous extracts.

**Table S1.** Compounds identified/tentatively identified in *Abies alba* and *Pinus sylvestris* bark aqueous extracts.

| **No.** | **T_R_**  **[min]** | **[M-H]^-^**  **[*m/z*]** | **MF** | **MS/MS fragments**  **[*m/z*]** | **Proposed**  **identity** | **Sample** | **Ref.** |
| --- | --- | --- | --- | --- | --- | --- | --- |
| **1** | 1.9 | 191.0601 | C_7_H_12_O_6_ | 173.0506, 127.0446, 109.0335, 85.0338, | quinic acid* | *P. sylvestris*  *A. alba* | [66] |
| **2** | 2.5 | 191.0247 | C_6_H_8_O_7_ | 129.0239, 111.0128, 87.0129 | citric acid* | *P. sylvestris*  *A. alba* | [67] |
| **3** | 3.8 | 299.0772 | C_13_H_16_O_8_ | 137.0226, 93.0334 | hydroxybenzoic acid-  *O*-hexoside | *P. sylvestris* | [25] |
| **4** | 7.0 | 329.0830 | C_14_H_18_O_9_ | 167.0425, 152.0186, 123.0506 | vanillic acid-*O*-hexoside | *P. sylvestris* | [67] |
| **5** | 10.7 | 577.1503 | C_30_H_26_O_12_ | 451.1016, 425.0912, 407.0766, 289.0715, 245.0767 | procyanidin dimer | *P. sylvestris* | [68,69] |
| **6** | 11.8 | 337.0954 | C_16_H_18_O_8_ | 191.0567, 163.0425 | coumaroylquinic acid I | *P. sylvestris* | [30] |
| **7** | 12.5 | 289.0778 | C_15_H_14_O_6_ | 245.0882, 205.0851, 151.0362, 125.0201 | catechin* | *P. sylvestris*  *A. alba* | [67,68,70] |
| **8** | 13.4 | 305.0699 | C_15_H_14_O_7_ | 261.0782, 219.0734, 167.0327, 137.0234, 125.0237, 109.0303 | gallocatechin | *A. alba* | [70] |
| **9** | 13.8 | 475.1811 | C_21_H_32_O_10_ | 313.1672, 151.0111 | monoterpene-di-*O*-hexoside | *A. alba* | [71] |
| **10** | 16.8 | 507.1896 | C_25_H_32_O_11_ | 327.1350, 315.1208, 300.1002, 181.0470 | unidentified | *A. alba* | - |
| **11** | 17.4 | 495.1900 | C_24_H_32_O_11_ | 363.1455, 345.1310, 327.1238, 315.1192, 179.0691, 167.0699 | unidentified | *P. sylvestris* | - |
| **12** | 18.0 | 289.0708 | C_15_H_14_O_6_ | 271.0666, 245.0733, 205.0654, 151.0311 | epicatechin* | *A. alba* | [67,70] |
| **13** | 18.1 | 337.0927 | C_16_H_18_O_8_ | 191.0567, 163.0425 | coumaroylquinic acid II | *P. sylvestris* | [30] |
| **14** | 19.5 | 525.1979 | C_25_H_34_O_12_ | 431.0992, 345.1404, 315.1235, 179.0754, 167.0733 | unidentified | *A. alba* | - |
| **15** | 20.3 | 491.1934 | C_25_H_32_O_10_ | 327.1266, 315.1228, 300.0999, 269.0780 | unidentified | *P. sylvestris* | - |
| **16** | 20.9 | 465.1066 | C_21_H_22_O_12_ | 303.0560, 285.0419, 259.0645, 152.0136, 125.0269 | taxifolin  hexoside | *P. sylvestris* | [68,72] |
| **17** | 21.0 | 491.1931 | C_25_H_32_O_10_ | 327.1255, 315.1219, 300.1006, 255.0621 | unidentified | *A. alba* | - |
| **18** | 21.7 | 479.2494 | C_22_H_40_O_11_ | 333.1925, 161.0413, 125.0237, 113.0224, 101.0247 | monoterpene-*O*-hexoside-  *O*-deoxyheoside | *P. sylvestris* | [71] |
| **19** | 22.2 | 521.1991 | C_26_H_34_O_11_ | 341.1363, 329.1397, 314.1204, 283.0876, 269.0784 | lariciresinol-*O*-hexoside | *A. alba* | [73] |
| **20** | 22.9 | 303.0512 | C_15_H_12_O_7_ | 285.0417, 259.0599, 217.0151, 175.0394, 151.0373, 125.0279 | taxifolin | *P. sylvestris* | [68,72] |
| **21** | 24.4 | 187.1015 | C_9_H_16_O_4_ | 169.0899, 143.1096, 125.1002 | azelaic acid | *P. sylvestris*  *A. alba* | [74] |
| **22** | 25.7 | 445.2101 | C_21_H_34_O_10_ | 313.1672, 161.0454, 113.0243 | monoterpene-*O*-hexoside-  *O*-pentoside | *P. sylvestris* | [71] |
| **23** | 25.9 | 473.2012 | C_21_H_34_O_11_ | 311.1533, 161.0454 | monoterpene-di-*O*-hexoside | *A. alba* | [71] |
| **24** | 26.6 | 459.2247 | C_22_H_36_O_10_ | 313.1613, 191.0524, 167.1063 | monoterpene-*O*-hexoside-  *O*-deoxyhexoside | *P. sylvestris*  *A. alba* | [71] |
| **25** | 27.5 | 447.2237 | C_21_H_36_O_10_ | 315.1783, 161.0508 | monoterpene-*O*-hexoside-  *O*-pentoside | *P. sylvestris* | [71] |
| **26** | 28.1 | 461.2370 | C_22_H_38_O_10_ | 315.1813, 161.0434 | monoterpene-*O*-hexoside-  *O*-deoxyhexoside | *P. sylvestris* | [71] |
| **27** | 28.9 | 201.1123 | C_10_H_18_O_4_ | 183.1052, 157.1238, 139.1166, 111.0849 | sebacic acid | *A. alba* | [75] |

MF, molecular formula; T_R_, retention time; * confirmed with the standard.

References

25. Karonen, M.; Hämäläinen, M.; Nieminen, R.; Klika, K.D.; Loponen, J.; Ovcharenko, V.V.; Moilanen, E.; Pihlaja, K. Phenolic extractives from the bark of *Pinus sylvestris* L. and their effects on inflammatory mediators nitric oxide and prostaglandin E_2_. *J. Agric. Food Chem*. **2004**, *52*, 7532-7540. doi: [10.1021/jf048948q](https://doi.org/10.1021/jf048948q)

30. Luca, S.V.; Miron, A.; Aprotosoaie, A.C.; Mihai, C.-T.; Vochita, G.; Gherghel, D.; Ciocarlan, N.; Skalicka-Woźniak, K. HPLC-DAD-ESI-Q-TOF-MS/MS profiling of *Verbascum ovalifolium* Donn ex Sims and evaluation of its antioxidant and cytogenotoxic activities. *Phytochem. Anal*. **2019**, *30*, 34-45. doi:[10.1002/pca.2788](http://dx.doi.org/10.1002/pca.2788)

66. Bujor, A.; Miron, A.; Luca, S.V.; Skalicka-Woźniak, K.; Silion, M.; Trifan, A.; Girard, C.; Demougeot, C.; Totoson, P. Vasorelaxant effects of *Crataegus pentagyna*: Links with arginase inhibition and phenolic profile. *J. Ethnopharmacol*. **2020**, *252*, 112559. doi:[10.1016/j.jep.2020.112559](https://doi.org/10.1016/j.jep.2020.112559)

67. Bujor, A.; Miron, A.; Luca, S.V.; Skalicka-Woźniak, K.; Silion, M.; Ancuceanu, R.; Dinu, M.; Girard, C.; Demougeot, C.; Totoson, P. Metabolite profiling, arginase inhibition and vasorelaxant activity of *Cornus mas*, *Sorbus aucuparia* and *Viburnum opulus* fruit extracts. *Food Chem. Toxicol*. **2019**, *133*, 110764. doi:[10.1016/j.fct.2019.110764](http://dx.doi.org/10.1016/j.fct.2019.110764)

68. Cretu, E.; Karonen, M.; Salminen, J.-P.; Mircea, C.; Trifan, A.; Charalambous, C.; Constantinou, A.I.; Miron, A. *In vitro* study on the antioxidant activity of a polyphenol-rich extract from *Pinus brutia* bark and its fractions. *J. Med. Food* **2013**, *16*, 984-991. doi:[10.1089/jmf.2013.0050](http://dx.doi.org/10.1089/jmf.2013.0050)

69. Karar, M.G.E.; Kuhnert, N. UPLC-ESI-Q-TOF-MS/MS characterization of phenolics from *Crataegus monogyna* and *Crataegus* *laevigata* (Hawthorn) leaves, fruits and their herbal derived drops (Crataegutt Tropfen). *J. Chem. Biol. Ther*. **2015**, *1,* 102. doi:[10.4172/2572-0406.1000102](http://dx.doi.org/10.4172/2572-0406.1000102)

70. Yuzuak, S., Ballington, J.; Xie, D.Y. HPLC-qTOF-MS/MS-based profiling of flavan-3-ols and dimeric proanthocyanidins in berries of two muscadine grape hybrids FLH 13-11 and FLH 17-66. *Metabolites* **2018**, *8*, 57. doi: [10.3390/metabo8040057](https://www.mdpi.com/2218-1989/8/4/57/htm)

71. ‘KNApSAcK’ Family. Available online: <http://www.knapsackfamily.com/KNApSAcK_Family/> (accessed on 21 December 2022)

72. Yang, C.-J.; Wang, Z.-B.; Mi, Y.-Y.; Gao, M.-J.; Lv, J.-N.; Meng, Y.-H.; Yang, B.-Y.; Kuang, H.-X. UHPLC-MS/MS determination, pharmacokinetic, and bioavailability study of taxifolin in rat plasma after oral administration of its nanodispersion. *Molecules* **2016**, *21*, 494. doi:[10.3390/molecules21040494](http://dx.doi.org/10.3390/molecules21040494)

73. Saber, F.R.; Mohsen, E.; El-Hawary, S.; Eltanany, B.M.; Elimam, H.; Sobeh, M.; Elmotayam, A.K. Chemometric-enhanced metabolic profiling of five *Pinus* species using HPLC-MS/MS spectrometry: Correlation to *in vitro* anti-aging, anti-Alzheimer and antidiabetic activities. *J. Chromatogr. B.* **2021**, *1177*, 122759. doi: [10.1016/j.jchromb.2021.122759](https://doi.org/10.1016/j.jchromb.2021.122759)

74. Zhang, X.; Liang, C.; Li, C.; Bu, M.; Bu, L.; Xiao, Y.; Sun, H.; Zhang, L. Simultaneous qualitative and quantitative study of main compounds in *Commelina communis* Linn. by UHPLC–Q-TOF-MS-MS and HPLC–ESI-MS-MS. *J. Chromatogr. Sci.* **2018**, *56*, 582-594. doi: [10.1093/chromsci/bmy030](https://doi.org/10.1093/chromsci/bmy030)

75. Rizzarelli, P.; Zampino, D.; Ferreri, L.; Impallomeni, G. Direct electrospray ionization mass spectrometry quantitative analysis of sebacic and terephthalic acids in biodegradable polymers. *Anal. Chem.* **2011**, *83*, 654-660. doi: [10.1021/ac102579q](https://doi.org/10.1021/ac102579q)

**Disclaimer/Publisher’s Note:** The statements, opinions and data contained in all publications are solely those of the individual author(s) and contributor(s) and not of MDPI and/or the editor(s). MDPI and/or the editor(s) disclaim responsibility for any injury to people or property resulting from any ideas, methods, instructions or products referred to in the content.
